# Invasive Fungal Infections after Anti-CD19 Chimeric Antigen Receptor-Modified T-Cell Therapy: State of the Evidence and Future Directions

**DOI:** 10.3390/jof7020156

**Published:** 2021-02-23

**Authors:** Will Garner, Palash Samanta, Ghady Haidar

**Affiliations:** 1 Division of Infectious Diseases, University of Pittsburgh Medical Center, Pittsburgh, PA 15213, USA; garnerw3@upmc.edu (W.G.); samantap@upmc.edu (P.S.); 2 Department of Medicine, University of Pittsburgh School of Medicine, Pittsburgh, PA 15213, USA

**Keywords:** CAR-T-cell therapy, risk factors, invasive fungal infection, prophylaxis, mold, yeast

## Abstract

Studies describing invasive fungal infections (IFIs) after chimeric antigen receptor-modified T-cell (CAR-T-cell) therapy are limited. Although post-CAR-T-cell IFIs appear to be uncommon, they are associated with significant morbidity and mortality. Specific risk factors for IFIs in CAR-T-cell recipients have not been fully characterized and are often extrapolated from variables contributing to IFIs in patients with other hematologic malignancies or those undergoing hematopoietic cell transplant. Optimal prophylaxis strategies, including the use of yeast versus mold-active azoles, also remain ill-defined. Further research should investigate key risk factors for IFIs and establish an evidence-based approach to antifungal prophylaxis in these patients in order to improve clinical outcomes.

## 1. Introduction

Chimeric antigen receptor-modified T-cell (CAR-T-cell) therapy targeting the B-cell antigen CD19 has drastically improved outcomes in patients with refractory B-cell malignancies [1,2,3,4,5]. However, managing the toxicities of CAR-T-cell therapies remains challenging. The two most common of these toxicities are the cytokine release syndrome (CRS) and the immune effector cell-associated neurotoxicity syndrome (ICANS) (formerly known as CAR-T-cell associated encephalopathy syndrome). These toxicities typically develop within the first 21 days of CAR-T-cell infusion during proliferation of CAR-T-cells. Treatment of CRS and ICANS may include the interleukin-6 inhibitor tocilizumab and/or corticosteroids depending on their severity (graded 1–4) [2]. Prolonged leukopenia (particularly lymphopenia) and hypogammaglobulinemia due to B-cell aplasia are also two direct CAR-T-cell toxicities and are generally thought to be mediated by “on-target, off-tumor” effects of CAR-T-cells, which occur when CAR-T-cells kill normal B-cells that express the CAR-T-cell target antigen [6]. Neutropenia may also be a direct toxicity of CAR-T-cell therapy, but its pathogenesis has not been fully defined [6,7].

Infections are among the indirect toxicities of CAR-T-cell therapy. CAR-T-cell recipients are at an increased risk of infection because of prior anti-neoplastic therapy, refractory malignancy, lymphodepleting conditioning chemotherapy (typically with fludarabine and cyclophosphamide), B-cell aplasia, the immune perturbations associated with CRS and ICANS, and their management with immunosuppressive therapies [8,9,10,11,12,13,14,15]. Nosocomial bacterial and respiratory viral infections are the most common infections after CAR-T-cell therapy. Invasive fungal infections (IFIs), in contrast, are uncommon, and studies providing detailed analyses of IFIs following CAR-T-cell therapy remain limited. Additionally, high-quality data informing antifungal prophylaxis practices are lacking.

Herein, we discuss the risk factors and epidemiology of post-CAR-T-cell IFIs. We also focus on areas that require further investigation, such as management algorithms and antifungal prophylaxis and monitoring. Throughout the manuscript, we will only be referring anti-CD19 CAR-T-cell products unless otherwise indicated.

## 2. Epidemiology of Fungal Infections after CAR-T-Cell Therapy

Seven published manuscripts and abstracts describing IFIs after CAR-T-cell therapy were identified at the time of this review [8,9,10,11,12,13,14]. Overall, IFIs after CAR-T-cell therapy are uncommon and have been reported in 1–15% of patients, with 0–10% and 0–7% of patients developing yeast and mold infections, respectively. Most IFIs occur within the first 30 days following CAR-T-cell therapy and typically represent breakthrough infections developing in patients receiving fluconazole or echinocandin prophylaxis. IFIs occurring >30 days after CAR-T-cell therapy, including invasive mold infections, have been described in patients with persistent risk factors such as prolonged neutropenia [8,9]. In one study which reported infections occurring >90 days after CAR-T-cell infusion, IFIs developed in 9% of patients and included two invasive mold infections, one yeast infection, and one endemic mycosis (*Coccidioides immitis* infection) [10]. Six studies reported infection-related deaths, of which mortality attributable to IFIs ranged from 0 to 5% [8,9,10,11,12,13].

### 2.1. Yeast Infections

Fourteen yeast infections in 13 unique patients following CAR-T-cell therapy have been reported [8,9,10,11,14,15] (Table 1). Seven of these episodes (50%) were cases of fungemia. Nine of the 14 yeast infections occurred within 30 days of CAR-T-cell infusion (early) including two *Candida glabrata* fungemias; the remainder were fungemias caused by *Candida tropicalis*, *Candida krusei*, and *Saccharomyces cerevisiae*. The additional early yeast infections described were two cases of respiratory tract infections attributed to *C. glabrata* and *Candida bracarensis*, one case of oropharyngeal candidiasis, and an intra-abdominal infection caused by *C. glabrata*. Of note, as true *Candida* respiratory tract infections are exceedingly uncommon in patients with hematological malignancies, the cases of *Candida* respiratory infections may have simply represented colonization. All patients who developed early yeast infections were receiving fluconazole prophylaxis, with the exception of the patient who developed the *S. cerevisiae* blood stream infection, who was receiving micafungin prophylaxis. Yeast infections >30 days after CAR-T-cell therapy were *C. glabrata* fungemia, oropharyngeal candidiasis, *Candida* esophagitis, and a case of *Candida albicans* fungemia with subsequent vertebral osteomyelitis. Notably, these patients were not receiving antifungal prophylaxis, but no specific IFI risk factors were described in the studies. Infection-related mortality was attributed to the *C. krusei* and *C. tropicalis* fungemias, both of which were early infections.

### 2.2. Mold Infections

Of the 15 invasive mold infections (IMIs) described after CAR-T-cell therapy, 11, 3, and 1 were proven, probable, and possible IMIs, respectively [8,9,10,11,12,13,14] (Table 2). Overall, the primary site of mold infection was the lung. Eight of the 15 IMIs occurred <30 days after CAR-T-cell infusion and included two *Aspergillus* species (spp.) infections, two Mucorales infections, two *Fusarium* spp. infections, an unidentified IMI, and one case of probable pulmonary aspergillosis. Of these early IMIs, 4, 3, and 1 patients were receiving fluconazole, micafungin, and voriconazole prophylaxis, respectively. The patient receiving voriconazole prophylaxis developed a Mucorales lung infection due to *Cunninghamella* spp., but had previously been diagnosed with probable pulmonary mold infection (without a positive culture) prior to CAR-T-cell therapy; it was therefore unclear whether the *Cunninghamella* infection was present prior to CAR-T-cell infusion, or whether it developed after therapy. Both *Fusarium* spp. infections were disseminated. One involved the central nervous system, and one (caused by *Fusarium solani*) was isolated from the patient’s thigh and sinuses; the latter infection developed while the patient was receiving fluconazole followed by posaconazole prophylaxis. IMIs occurring >30 days after CAR-T-cell therapy included three *Aspergillus* spp. infections, one Mucorales infection, one case each of probable and possible invasive mold infection, and a skin and soft tissue infection from which both an *Aspergillus* and *Rhizopus* spp. were identified. Four of 15 (27%) patients who developed IMI died from their infection, three of whom died within 30 days of CAR-T-cell infusion. In one study describing three IMIs, two early infections occurred in patients who developed severe CRS/ICANS requiring tocilizumab +/− corticosteroids, and the single late IMI occurred in the setting of persistent disease and prolonged neutropenia [8]. Additionally, the central nervous system *Fusarium* spp. infection occurred after the administration of a long course of steroids. Three of the studies reporting IMIs did not describe predisposing patient risk factors.

### 2.3. Other Fungal Infections

Non-yeast and non-mold IFIs after CAR-T-cell therapy are extremely uncommon. In the literature, three cases of *Pneumocystis jirovecii* pneumonia (PCP) and one case of coccidioidomycosis have been reported [8,9,10,13] (Table 3). Two of the PCP cases occurred >90 days after CAR-T-cell therapy. One patient developed PCP nine months after CAR-T-cell therapy, with a CD4^+^ count of 44 cells/μL at the time of infection. Pentamidine prophylaxis had been discontinued four months prior. A second patient was also diagnosed with PCP nine months after CAR-T-cell therapy and had an absolute lymphocyte count of 100 cells/μL (CD4^+^ count not available) at the time of infection; trimethoprim/sulfamethoxazole prophylaxis had been discontinued 3 months prior. The third case of PCP occurred between within 3 months of CAR-T-cell infusion in the setting of trimethoprim/sulfamethoxazole prophylaxis non-compliance. The *Coccidioides* infection occurred >1 year after CAR-T-cell infusion in the Pacific Northwestern United States, although no further demographic or prophylaxis data were available for this patient. One of the patients who developed PCP died of a subsequent bacterial infection, and outcome data for the other two fungal infections were not available.

## 3. Risk Factors for Fungal Infections after CAR-T-Cell Therapy

Because only a few studies describing infections after CAR-T-cell therapy have been published [8,9,10,11,12,13,14,15], CAR-T-cell-specific risk factors for IFI remain ill-defined. Thus, factors that increase the risk for other infections after CAR-T-cell therapy and variables known to be associated with an increased risk of IFIs in other patients with hematological malignancies can be used to evaluate the IFI risk of a CAR-T-cell therapy recipient instead. For instance, risk factors predisposing patients to infections after CAR-T-cell therapy include pre-CAR-T-cell variables such as prior HCT, number of prior lines of chemotherapy, CAR-T-cell dose, acute lymphoblastic leukemia, and history of infection prior to CAR-T-cell therapy [8,9,10], as well as post-CAR-T-cell variables such as higher grades of CRS (≥grade 3) and potentially neutropenia and the use of tocilizumab or steroids [11]. While it is biologically plausible for these variables to be associated with an increased risk of IFI specifically, additional studies are needed.

Major risk factors for IFI in patients with hematologic malignancies and those undergoing HCT include neutropenia, steroid use, indwelling central venous catheters (CVCs), oral/gastrointestinal tract mucositis after induction chemotherapy, intensive care unit (ICU) stay, refractory disease, previous history of IFI, and malignancy type—specifically acute myelogenous leukemia (AML) or high-risk myelodysplastic syndrome [16,17,18,19,20,21,22]. Many of these risk factors, such as prolonged cytopenias, steroid use, CVCs, refractory malignancy, and prior history of IFI, are common in CAR-T-cell recipients (Table 4). Leukopenia occurring after CAR-T-cell therapy warrants special mention. Indeed, neutropenia and lymphopenia persisting well beyond the duration expected after conditioning chemotherapy have been described, and both likely contribute to a durable risk of IFI after CAR-T-cell therapy [20,21,22]. As fludarabine and cyclophosphamide, the most common conditioning chemotherapy used prior to CAR-T-cell therapy, do not typically cause severe mucositis, the risk of early *Candida* infections is expected to be low. Nonetheless, invasive candidiasis has been described in 0–3% of patients in the first 30 days after CAR-T-cell infusion despite the use of appropriate prophylaxis, which may be a result of a heightened state of immunosuppression related to other early CAR-T-cell related toxicities, such as CRS and ICANS [8,9,11,12,13,14]. It is unclear whether tocilizumab use is associated with an increased risk of IFI in these patients. Although pooled data of phase-three trials of tocilizumab for rheumatoid arthritis suggested an increased risk of IFI [23], the absolute risk was low, with 10 IFIs reported among 4000 patients. Additionally, the median duration of tocilizumab therapy in these trials was 2.4 years with monthly dosing, whereas CAR-T-cell recipients receive only a few doses in the early weeks after CAR-T-cell therapy.

How do we synthesize the above data and apply them to CAR-T-cell therapy recipients specifically? Based on the above, it appears rational to conclude that a combination of pre-infusion factors (e.g., extent of prior chemotherapy including HCT, type of malignancy, previous history of IFI) and post-infusion factors (e.g., neutropenia, lymphopenia, steroid exposure, ICU admission, and presence of indwelling CVCs) may increase the risk of IFI. Additionally, the type of underlying malignancy is likely to have a significant influence on the risk of IFI after CAR-T-cell therapy, as risk of IFI is greater in patients with acute lymphoblastic leukemia (ALL) compared with those with diffuse large B-cell lymphoma and chronic lymphocytic leukemia [24]. However, current studies have either included only a single type of malignancy or were not sufficiently powered enough to detect differences in risk between different malignancy types. Identifying risk of IFI by malignancy type will become increasingly important as the breadth of CAR-T-cell indications increases. Whether the timing of CAR-T-cell therapy impacts the risk of IFI has not been fully studied, although the number of lines of chemotherapy prior to CAR-T-cell infusion and history of HCT have been associated with overall infection risk [8,9]. Thus, patients who receive CAR-T-cells early after their cancer diagnosis may be at a lower risk for IFI because of an overall lower net state of immunosuppression compared with those who have undergone multiple lines of chemotherapy prior to CAR-T-cell infusion. 

Risk factors for early *versus* late IFI may also differ. Because universal antifungal prophylaxis is the most common strategy used by most cancer centers, early IFIs are expected to represent breakthrough infections, both in patients whose pre-CAR-T-cell antifungals were continued after CAR-T-cell therapy, and those who were only initiated on antifungals at the time of the pre-CAR-T-cell conditioning regimen. In contrast, late IFIs, particularly those developing after prophylaxis is discontinued, are more likely to represent the true natural risk for IFI.

## 4. Anti-Fungal Prophylaxis Following CAR-T-Cell Therapy

### 4.1. Yeast Versus Mold-Active Prophylaxis

Because risk factors for IFIs in patients receiving CAR-T-cell therapy are not well-defined, there is no consensus about the optimal choice and duration of antifungal prophylaxis after CAR-T-cell therapy. As such, clinical practice varies widely among different centers. Although anti-yeast prophylaxis during the period of neutropenia after CAR-T-cell therapy has been the most commonly used strategy in clinical trials [3,4,5,9], it is not currently known whether certain subgroups of CAR-T-cell recipients may benefit from anti-mold prophylaxis. Indeed, there is much controversy around the optimal approach of yeast-versus-mold-active prophylaxis in these patients. Proposed strategies have included universal yeast-active prophylaxis, a tiered “risk stratification” approach, universal anti-mold prophylaxis, and pre-emptive therapy using fungal biomarkers and radiographic imaging [25,26,27]. At the center of the controversy is the absence of trials demonstrating whether anti-mold prophylaxis confers any mortality benefit in this population. Thus, robust evidence-based guidelines for antifungal prophylaxis such as those outlined by the European Conference on Infections in Leukemia [28] for other hematological malignancy patients do not currently exist.

Nonetheless, in the past few years, several guidance documents have been published with provisional suggestions about the optimal approach to antifungal prophylaxis in these patients. Recent CAR-T-cell therapy expert panel guidelines suggest fluconazole or micafungin prophylaxis against *Candida* during neutropenia [1]. Another guideline from the European Society for Blood and Marrow Transplantation recommends mold-active azole prophylaxis in patients with prior allogenic HCT, prior invasive aspergillosis, and those receiving corticosteroids [29]. Other groups have suggested that ≥4 prior anti-tumor treatment lines, CAR-T-cell dose of >2 × 10^7^/kg, prolonged neutropenia (≥3 weeks), and use of >1 dose of tocilizumab or the administration of other immunosuppressive agents (such as anakinra and siltuximab) for the management of CRS and ICANS should also warrant the use of mold-active antifungal prophylaxis [30,31].

**Figure 1:**
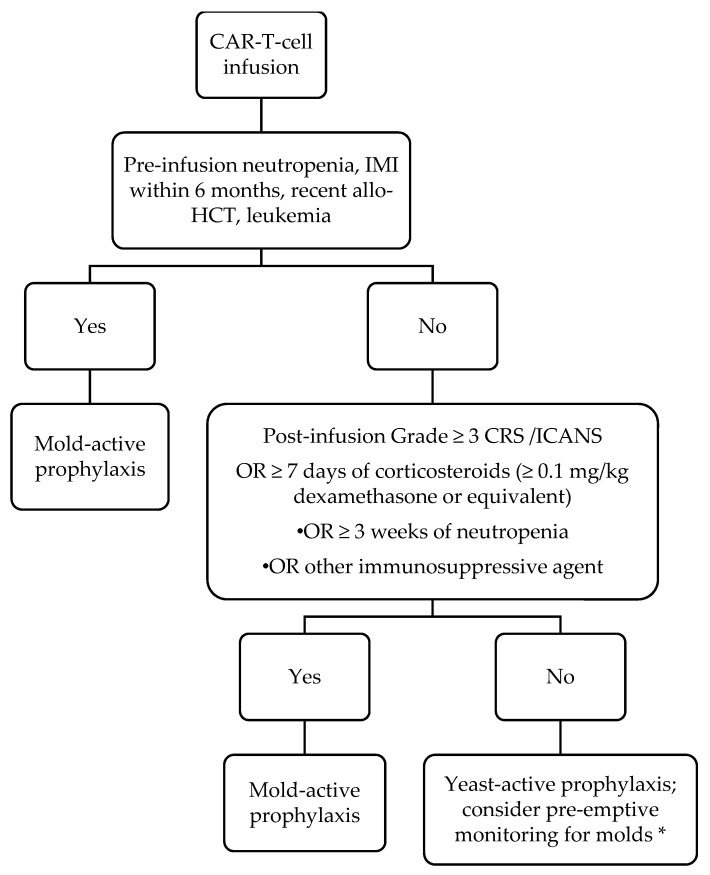
Our approach to anti-fungal prophylaxis for prevention of invasive fungal infection post-chimeric antigen receptor-modified T-cell (CAR T-cell) therapy. Abbreviations: IMI = invasive mold infection; allo-HCT = allogeneic hematopoietic cell transplantation; CRS = cytokine release syndrome; ICANS = immune effector cell-associated neurotoxicity syndrome. For our purposes, we define neutropenia as an absolute neutrophil count of ≤500/µL. Duration of mold-active prophylaxis should be individualized. We maintain patients on mold-active agents until at least 1 month after discontinuation of immunosuppression AND resolution of neutropenia. Posaconazole is our preferred agent; voriconazole and isavuconazole are reasonable alternatives based on side effect profile and cost. * Pre-emptive therapy consists of diagnostics such as fungal biomarkers (serum beta-D-glucan, galactomannan) and surveillance radiographic imaging.

Based on the current literature and extrapolation from risk factors for mold infections in other hematological malignancy patients, we have adopted the antifungal prophylaxis protocol described in Figure 1, which generally classifies patients as “high risk” for mold infection based on whether they are “AML-like” due to the presence of prolonged neutropenia, or “graft-versus-host disease (GVHD)-like” due to the use of corticosteroids or other immunosuppressive agents. We use posaconazole as our preferred mold-active prophylactic agent because of clinical trial data supporting its use in patients with AML and GVHD [32,33]. However, until data in CAR-T-cell therapy recipients are generated, we believe that any mold-active antifungal (such as voriconazole or isavuconazole) may be acceptable, and that the specific choice of agent should be guided by history of prior mold infection, side effect profile (e.g., avoidance of voriconazole in persons with neurotoxicity), and cost. Although this is our approach, others have advocated universal mold-active prophylaxis because of the uncertainties surrounding risk factors for mold infection after CAR-T-cell therapy. Specific concerns that were cited include the risks of mold infection in treatment-experienced ALL patients and challenges predicting duration of cytopenias and extent of steroid exposure [26].

There are no data to guide the duration of mold-active prophylaxis. Although the paradigm in Figure 1 outlines a general framework for duration depending on the presence of neutropenia and the use of steroids, the precise duration of prophylaxis should be determined on a case-by-case basis based on the resolution of risk factors. A pre-emptive approach relying on biomarkers and imaging [34] has not been validated in these patients and may be hampered by limited testing availability and slow turnaround times. While the CD4^+^ T-cell cell count is an appealing marker that may help guide and individualize the duration of mold-active or other antifungal prophylaxis, further research validating this approach would need to be conducted prior to widespread inclusion of CD4^+^ T-cell. measurements in prophylactic algorithms. Ultimately, there is a need to conduct large multicenter prospective studies, preferably randomized clinical trials similar to the pivotal trials of posaconazole in AML and GVHD [32,33], to determine the benefit of yeast versus mold-active antifungal prophylaxis in CAR-T-cell therapy recipients.

### 4.2. Prophylaxis Against PCP

It is standard practice to administer trimethoprim-sulfamethoxazole (or alternatives, such as dapsone, atovaquone, and monthly intravenous pentamidine) for 3–6 months after CAR-T-cell therapy to prevent PCP [6,30,31]. Given that many CAR-T-cell patients are expected to experience prolonged lymphopenia due to “on-target, off-tumor” effects of CAR-T-cells, these patients may be at risk for PCP beyond 6 months. Some authors have suggested that PCP prophylaxis be continued until the CD4^+^ T-cell count is greater than 200 cells/µL [30]. Indeed, cases of PCP have been reported over 6 months after CAR-T-cell therapy in lymphopenic patients whose PCP prophylaxis had been discontinued [8,9,13]. Based on these data, we currently recommend at least 1 year of anti-PCP prophylaxis at our center, which can be stopped once the CD4^+^ T-cell count is greater than 200 cells/µL.

## 5. Our Approach to Work-Up and Management of Fungal Infections after CAR-T-Cell Therapy

When caring for CAR-T-cell therapy recipients with suspected IFIs, we recommend following the same guidelines that have been established for the diagnosis and management of IFIs in other patients with hematologic malignancies [35]. However, there are some nuances to consider. Part of the traditional approach for managing hematological malignancy patients with neutropenic fever includes treating and evaluating for IFIs if fevers do not resolve after around 5 days of appropriate antibacterial therapy. However, severe neutropenia is common after CAR-T-cell therapy, and fever occurs in up to 92% of recipients, secondary to CRS and/or infection [1]. Thus, persistent fevers in a neutropenic CAR-T-cell recipient may simply be due to ongoing CRS as opposed to an occult infection, suggesting that an urgent evaluation for mold infections may not be necessary in persistently febrile CAR-T-cell therapy recipients. Unfortunately, there are no clinical or laboratory characteristics that can reliably distinguish between CRS and infections, although early studies suggest a possible role of relying on cytokine signatures [36]. Nonetheless, neutropenic fever should be managed with a detailed history and physical examination, blood cultures, and symptom-driven radiographic imaging. Other approaches, such as surveillance chest CT imaging, pre-emptive screening for fungal biomarkers such as the galactomannan assay [34], and empirical use of mold-active antifungal therapy in patients with persistent fevers, warrant further study.

## 6. Conclusions

In conclusion, IFIs in the post-CAR-T-cell period, while uncommon, contribute to significant morbidity and mortality. Because of the limited studies of IFIs after CAR-T-cell therapy, risk factors for these infections are extrapolated from other patient populations. Most IFIs are caused by *Candida* and molds, although three cases of PCP were reported within the first year after CAR-T-cell therapy in the setting of prophylaxis discontinuation. Guidelines have suggested providing prophylaxis against *Candida* during periods of neutropenia, which is generally accepted practice. Less consensus regarding when to initiate mold-active prophylaxis exists, resulting in varied guidelines and institutional-specific practices. Ultimately, further studies are warranted to better describe the epidemiology of IFI after CAR-T-cell therapy. Large multicenter prospective studies are necessary to establish best practices for prevention and management of IFIs in this vulnerable population.

## Figures and Tables

**Table 1:** Published reports of invasive yeast infections following chimeric antigen receptor-modified T-cell (CAR-T-cell) therapy. Neutropenia defined as absolute neutrophil count <500 cells/μL. Lymphopenia defined as absolute lymphocyte count <1000 cells/μL. ALL = acute lymphoblastic leukemia; DLBCL = diffuse large B-cell lymphoma; CRS = cytokine release syndrome; ICANS = immune effector cell-associated neurotoxicity syndrome. Dashes indicate that the data were not reported in the studies. ^a^ As true invasive *Candida* spp. respiratory tract infections are rare in patients with hematological malignancies, it is unclear if these isolates represent invasive infections or simply colonization.

Ref.	Fungal Infection	Cancer	Prophylaxis	Neutropenia	Lymphopenia	Time of Onset of Infection	CRS	Steroids	Tocilizumab Given?	Previous Transplant	Died of Fungal Infection?
Park et al. [11]	*Saccharomyces cerevisiae*: fungemia	ALL	Micafungin	Yes	–	Day 0–30	Grade 3	–	–	–	No
Garner et al. [8]	*Candida tropicalis*: fungemia	DLBCL	Fluconazole	Yes	Yes	Day 0–30	Grade 2	Yes	Yes (2 doses)	No	Yes
	*Candida glabrata*: intra-abdominal infection	DLBCL	Fluconazole	No	Yes	Day 0–30	Grade 1	Yes	Yes (1 dose)	Yes (autologous)	No
	*Candida* esophagitis	DLBCL	Fluconazole	No	Yes	Day 0–30	Grade 2	Yes	Yes (1 dose)	Yes (autologous)	No
	*Candida albicans:* fungemia	DLBCL	None	No	Yes	Day 30+	Grade 2	No	Yes (1 dose)	Yes (autologous)	No
	*Candida albicans*: vertebral osteomyelitis	DLBCL	None	–	–	Day 30+	Grade 2	No	Yes (1 dose)	Yes (autologous)	No
	*Candida* esophagitis	DLBCL	None	No	Yes	Day 30+	No	No	No	No	No
Hill et al. [9]	*Candida glabrata:* fungemia	–	Fluconazole	–	–	Day 0–30	–	–	–	–	No
	*Candida glabrata:* fungemia	–	Fluconazole	–	–	Day 0–30	–	–	–	–	No
	*Candida glabrata:* lungs ^a^	–	Fluconazole	–	–	Day 0–30	–	–	–	–	No
	*Candida bracarensis:* lungs ^a^	–	Fluconazole	–	–	Day 0–30	–	–	–	–	No
Tran et al. [15]	*Candida glabrata*: fungemia	–	–	–	–	Day 30+	–	–	–	–	–
Cordeiro et al. [10]	Oral candidiasis	–	–	–	–	Day 30+	–	–	–	–	No
Louge et al. [14]	*Candida krusei* fungemia	DLBCL	Fluconazole	–	–	38 days	–	Yes, for ICANS	–	No	Yes

**Table 2:** Published reports of invasive mold infections following CAR-T-cell therapy. Neutropenia defined as absolute neutrophil count <500 cells/μL. Lymphopenia defined as absolute lymphocyte count <1000 cells/μL. ALL = acute lymphoblastic leukemia; BAL = bronchoalveolar lavage; CLL = chronic lymphocytic leukemia; CRS = cytokine release syndrome; DLBCL = diffuse large B-cell lymphoma; ICANS = immune effector cell-associated neurotoxicity syndrome. Dashes indicate that the data were not reported in the studies.

Ref.	Fungal Infection	Cancer	Prophylaxis	Neutropenia	Lymphopenia	Time of Onset of Infection	CRS	Steroids	Tocilizumab Given?	Previous Transplant	Died of Fungal Infection?
Park et al. [11]	*Aspergillus fumigatus*: pulmonary	ALL	Micafungin	Yes	–	Day 0–30	Grade 3	–	–	–	Yes
	Probable pulmonary aspergillosis (+BAL galactomannan)	ALL	Micafungin	Yes	–	Day 0–30	Grade 1	–	–	–	No
	Mucormycosis: lung	ALL	Micafungin	Yes	–	Day 0–30	No	–	–	–	No
	Probable pulmonary aspergillosis (+serum galactomannan)	ALL	None	Yes	–	Day 30+	–	–	–	–	No
Garner et al. [8]	*Fusarium solani;* skin and sinuses	ALL	Fluconazole → posaconazole	Yes	Yes	Day 0–30 possibly pre-infusion	Grade 1	No	Yes (3 doses)	No	No
	Mucorales; sinuses	Hairy cell leukemia	Voriconazole	Yes	Yes	Day 30+	Grade 1	No	Yes (1 dose)	Yes (allogeneic)	Yes
	Possible pulmonary aspergillosis	CLL	None	Yes	No	Day 30+	None	No	No	No	No
Hill et al. [9]	*Aspergillus ustus;* lungs	CLL	Fluconazole	No	–	Day 0–30	≥Grade 3	–	–	–	Yes
	Unknown mold; sinuses	ALL	Fluconazole	–	–	Day 0–30; possibly pre-infusion	≥Grade 3	–	–	–	No
	*Aspergillus fumigatus*; sinuses	CLL	Fluconazole	Yes	–	Day 30+	≥Grade 3	–	–	Yes (allogeneic)	No
Tran et al. [15]	*Aspergillus + Rhizopus species*: skin and soft tissue	–	–	–	–	Day 30+	–	-–	–	–	–
Cordeiro et al. [10]	Aspergillosis (*n* = 2)	–	–	–	–	Day 30+	–	–	–	–	No
Logue et al. [14]	Disseminated fusariosis	DLBCL	Fluconazole → micafungin	–	–	Day 0–30	–	Yes, for ICANS	–	No	Yes
Vora et al. [12]	*Cunninghamella species:* lung	ALL	Voriconazole	Yes	–	Day 0–30; possibly pre-infusion	Grade 1	No	Yes	No	Yes

**Table 3:** Published reports of other invasive fungal infections following CAR-T-cell therapy. Neutropenia defined as absolute neutrophil count <500 cells/μL. Lymphopenia defined as absolute lymphocyte count <1000 cells/μL. ALL = acute lymphoblastic leukemia; DLBCL = diffuse large B-cell lymphoma; CRS = cytokine release syndrome; TMP/SMX = trimethoprim/sulfamethoxazole; PCP = *Pneumocystis jirovecii* pneumonia. Dashes indicate that the data were not reported in the studies.

Ref.	Fungal Infection	Cancer	Prophylaxis	Neutropenia	Lymphopenia	Time of Onset of Infection	CRS	Steroids	Tocilizumab Given?	Previous Transplant	Died of Fungal Infection?
Garner et al. [8]	PCP	DLBCL	None (completed TMP/SMX)	No	Yes	Day 30+	Grade 2	Yes	Yes (2 doses)	No	No
Hill et al. [9]	PCP	ALL	None (TMP/SMX non-compliance)	–	–	Day 30+	–	–	–	–	No
Wudhikarn et al. [13]	PCP	–	None (completed pentamidine)	–	Yes	Day 30+	–	–	–	–	No
Cordeiro et al. [10]	*Coccidioides* infection	–	–	–	–	Day 30+	–	–	–	–	No

**Table 4:** Known and proposed risk factors for general infection and invasive fungal infections after CAR-T-cell therapy. HCT = hematopoietic cell transplant; CAR-T-cell = chimeric antigen receptor-modified T-cell; ALL = acute lymphoblastic leukemia; CRS = cytokine release syndrome; IFI = invasive fungal infection; CVCs = central venous catheters; CLL = chronic lymphocytic leukemia; DLBCL = diffuse large B-cell lymphoma; ICANS = immune effector cell-associated neurotoxicity syndrome. ^a^ Other potential, but less well-studied IFI risk factors include malignancy type (ALL vs. CLL vs. DLBCL), tocilizumab (number of doses), and use of alternative immunosuppressing agents in treatment of CRS or ICANS = immune effector cell-associated neurotoxicity syndrome.

	Risk Factors for Any Infection after CAR-T-Cell Therapy	Other Potential Risk Factors for IFI after CAR-T-Cell Therapy ^a^
Pre-Infusion Factors	Prior history HCT # of prior lines of chemotherapy CAR-T-cell dose ALL History of infection prior to CAR-T-cell therapy	Refractory disease/# of prior lines of chemotherapy Type of underlying malignancy Prior history of HCT Previous history of IFI Indwelling CVCs
Post-Infusion Factors	Higher CRS grade (≥3)	Neutropenia Lymphopenia Steroids (dose/duration) ICU admission Indwelling CVCs
Potential Additional Post-Infusions Factors	Neutropenia Lymphopenia Tocilizumab (# of doses) Steroids (dose/duration) Use of alternative immunosuppressing agents in treatment of CRS or ICANS	

## References

[1] Kansagra A.J., Frey N.V., Bar M., Laetsch T.W., Carpenter P.A., Savani B.N., Heslop H.E., Bollard C.M., Komanduri K.V., Gastineau D.A. (2019). Clinical Utilization of Chimeric Antigen Receptor T-Cells (Car-T) in B-Cell Acute Lymphoblastic Leukemia (All)—An Expert Opinion from the European Society for Blood and Marrow Transplantation (Ebmt) and the American Society for Blood and Marrow Transplantation (Asbmt). Bone Marrow Transplant..

[2] Mahadeo K.M., Khazal S.J., Abdel-Azim H., Fitzgerald J.C., Taraseviciute A., Bollard C.M., Tewari P., Duncan C., Traube C., McCall D. (2019). Management guidelines for paediatric patients receiving chimeric antigen receptor T cell therapy. Nat. Rev. Clin. Oncol..

[3] Maude S.L., Laetsch T.W., Buechner J., Rives S., Boyer M., Bittencourt H., Bader P., Verneris M.R., Stefanski H.E., Myers G.D. (2018). Tisagenlecleucel in Children and Young Adults with B-Cell Lymphoblastic Leukemia. N. Engl. J. Med..

[4] Neelapu S.S., Locke F.L., Bartlett N.L., Lekakis L.J., Miklos D.B., Jacobson C.A., Braunschweig I., Oluwole O.O., Siddiqi T., Lin Y. (2017). Axicabtagene Ciloleucel CAR T-Cell Therapy in Refractory Large B-Cell Lymphoma. N. Engl. J. Med..

[5] June C.H., Sadelain M. (2018). Chimeric Antigen Receptor Therapy. N. Engl. J. Med..

[6] Haidar G., Garner W., Hill J.A. (2020). Infections after Anti-Cd19 Chimeric Antigen Receptor T-Cell Therapy for Hematologic Malignancies: Timeline, Prevention, and Uncertainties. Curr. Opin. Infect. Dis..

[7] Fried S., Avigdor A., Bielorai B., Meir A., Besser M.J., Schachter J., Shimoni A., Nagler A., Toren A., Jacoby E. (2019). Early and late hematologic toxicity following CD19 CAR-T cells. Bone Marrow Transplant..

[8] Garner W., Samanta P., Dorritie K., Sehgal A., Winfield D., Agha M., Boudreau R., Nguyen M.H.T., Haidar G. (2020). 1105. The Burden of Infections Prior to Chimeric Antigen Receptor (CAR) Modified T-cell Therapy Predicts Post-CAR T-cell Infectious Complications. Open Forum Infect. Dis..

[9] Hill J.A., Li D., Hay K.A., Green M.L., Cherian S., Chen X., Riddell S.R., Maloney D.G., Boeckh M., Turtle C.J. (2018). Infectious complications of CD19-targeted chimeric antigen receptor–modified T-cell immunotherapy. Blood.

[10] Cordeiro A., Bezerra E.D., Hirayama A.V., Hill J.A., Wu Q.V., Voutsinas J., Sorror M.L., Turtle C.J., Maloney D.G., Bar M. (2020). Late Events after Treatment with CD19-Targeted Chimeric Antigen Receptor Modified T Cells. Biol. Blood Marrow Transplant..

[11] Park J.H., Romero F.A., Taur Y., Sadelain M., Brentjens R.J., Hohl T.M., Seo S.K. (2018). Cytokine Release Syndrome Grade as a Predictive Marker for Infections in Patients With Relapsed or Refractory B-Cell Acute Lymphoblastic Leukemia Treated With Chimeric Antigen Receptor T Cells. Clin. Infect. Dis..

[12] Vora S.B., Waghmare A., A Englund J., Qu P., A Gardner R., A Hill J. (2020). Infectious Complications Following CD19 Chimeric Antigen Receptor T-cell Therapy for Children, Adolescents, and Young Adults. Open Forum Infect. Dis..

[13] Wudhikarn K., Palomba M.L., Pennisi M., Garcia-Recio M., Flynn J.R., Devlin S.M., Afuye A., Silverberg M.L., Maloy M.A., Shah G.L. (2020). Infection during the first year in patients treated with CD19 CAR T cells for diffuse large B cell lymphoma. Blood Cancer J..

[14] Logue J.M., Zucchetti E., Bachmeier C.A., Krivenko G.S., Larson V., Ninh D., Grillo G., Cao B., Kim J., Chavez J.C. (2020). Immune reconstitution and associated infections following axicabtagene ciloleucel in relapsed or refractory large B-cell lymphoma. Haematologica.

[15] Tran N., Eschenauer G., Scappaticci G., Frame D., Miceli M., Patel T. (2020). Infections in Patients Treated with Chimeric Antigen Receptor T-Cells (Car-T) Therapy. Open Forum Infect. Dis..

[16] Wingard J.R., Leather H.L. (2001). Empiric antifungal therapy for the neutropenic patient. Oncology (Williston Park).

[17] Lortholary O., Gangneux J.-P., Sitbon K., Lebeau B., De Monbrison F., Le Strat Y., Coignard B., Dromer F., Bretagne S. (2011). Epidemiological trends in invasive aspergillosis in France: The SAIF network (2005–2007). Clin. Microbiol. Infect..

[18] Pagano L., Akova M., Dimopoulos G., Herbrecht R., Drgona L., Blijlevens N. (2010). Risk assessment and prognostic factors for mould-related diseases in immunocompromised patients. J. Antimicrob. Chemother..

[19] Wingard J.R. (2005). The changing face of invasive fungal infections in hematopoietic cell transplant recipients. Curr. Opin. Oncol..

[20] Wingard J. (1999). Fungal infections after bone marrow transplant. Biol. Blood Marrow Transplant..

[21] Tisi M.C., Hohaus S., Cuccaro A., Innocenti I., De Carolis E., Za T., D’Alò F., Laurenti L., Fianchi L., Sica S. (2016). Invasive fungal infections in chronic lymphoproliferative disorders: A monocentric retrospective study. Haematologica.

[22] Garcia-Vidal C., Upton A., Kirby K.A., Marr K.A. (2008). Epidemiology of Invasive Mold Infections in Allogeneic Stem Cell Transplant Recipients: Biological Risk Factors for Infection According to Time after Transplantation. Clin. Infect. Dis..

[23] Schiff M.H., Kremer J.M., Jahreis A., Vernon E., Isaacs J.D., Van Vollenhoven R.F. (2011). Integrated safety in tocilizumab clinical trials. Arthritis Res. Ther..

[24] Pagano L., Caira M., Candoni A., Offidani M., Fianchi L., Martino B., Pastore D., Picardi M., Bonini A., Chierichini A. (2006). The epidemiology of fungal infections in patients with hematologic malignancies: The SEIFEM-2004 study. Haematologica.

[25] Haidar G., Dorritie K., Farah R., Bogdanovich T., Nguyen M.H., Samanta P. (2019). Invasive Mold Infections After Chimeric Antigen Receptor–Modified T-Cell Therapy: A Case Series, Review of the Literature, and Implications for Prophylaxis. Clin. Infect. Dis..

[26] E Lewis R., Kontoyiannis D.P. (2020). Chimeric Antigen Receptor T-cell Immunotherapy and Need for Prophylaxis for Invasive Mold Infections. Clin. Infect. Dis..

[27] Haidar G., Nguyen M.H., Samanta P. (2020). Reply to Lewis and Kontoyiannis. Clin. Infect. Dis..

[28] A Maertens J., Girmenia C., Brüggemann R.J., Duarte R.F., Kibbler C.C., Ljungman P., Racil Z., Ribaud P., A Slavin M., A Cornely O. (2018). European guidelines for primary antifungal prophylaxis in adult haematology patients: Summary of the updated recommendations from the European Conference on Infections in Leukaemia. J. Antimicrob. Chemother..

[29] Yakoub-Agha I., Chabannon C., Bader P., Basak G.W., Bonig H., Ciceri F., Corbacioglu S., Duarte R.F., Einsele H., Hudecek M. (2019). Management of adults and children undergoing chimeric antigen receptor T-cell therapy: Best practice recommendations of the European Society for Blood and Marrow Transplantation (EBMT) and the Joint Accreditation Committee of ISCT and EBMT (JACIE). Haematologica.

[30] Los-Arcos I., Iacoboni G., Aguilar-Guisado M., Alsina-Manrique L., De Heredia C.D., Fortuny-Guasch C., García-Cadenas I., García-Vidal C., González-Vicent M., Hernani R. (2020). Recommendations for screening, monitoring, prevention, and prophylaxis of infections in adult and pediatric patients receiving CAR T-cell therapy: A position paper. Infection.

[31] Hill J.A., Seo S.K. (2020). How I prevent infections in patients receiving CD19-targeted chimeric antigen receptor T cells for B-cell malignancies. Blood.

[32] Ullmann A.J., Lipton J.H., Vesole D.H., Chandrasekar P., Langston A., Tarantolo S.R., Greinix H., De Azevedo W.M., Reddy V., Boparai N. (2007). Posaconazole or Fluconazole for Prophylaxis in Severe Graft-versus-Host Disease. N. Engl. J. Med..

[33] Cornely O.A., Maertens J., Winston D.J., Perfect J., Ullmann A.J., Walsh T.J., Helfgott D., Holowiecki J., Stockelberg D., Goh Y.-T. (2007). Posaconazole Vs. Flu-conazole or Itraconazole Prophylaxis in Patients with Neutropenia. N. Engl. J. Med..

[34] Maertens J., Theunissen K., Verhoef G., Verschakelen J., Lagrou K., Verbeken E., Wilmer A., Verhaegen J., Boogaerts M., Van Eldere J. (2005). Galactomannan and computed tomography-based preemptive antifungal therapy in neutropenic patients at high risk for invasive fungal infection: A prospective feasibility study. Clin. Infect. Dis..

[35] Freifeld A.G., Bow E.J., Sepkowitz K.A., Boeckh M.J., Ito J.I., Mullen C.A., Raad I.I., Rolston K.V., Young J.H., Wingard J.H. (2011). Clinical practice guideline for the use of antimicrobial agents in neutropenic patients with cancer: 2010 update by the infectious diseases society of america. Clin. Infect. Dis..

[36] Luo H., Wang N., Huang L., Zhou X., Jin J., Li C., Wang D., Xu B., Xu J., Jiang L. (2019). Inflammatory signatures for quick diagnosis of life-threatening infection during the CAR T-cell therapy. J. Immunother. Cancer.

